# Artificial Intelligence-Guided Artificial Nutrition in Critical Illness: Integrating Indirect Calorimetry and BIVA for Metabolic Precision

**DOI:** 10.3390/nu18091387

**Published:** 2026-04-28

**Authors:** Marialaura Scarcella, Antonella Cotoia, Luigi Vetrugno, Emidio Scarpellini, Gian Marco Petroni, Cristian Deana, Rachele Simonte, Riccardo Monti, Rita Commissari, Edoardo De Robertis, Elena Bignami

**Affiliations:** 1 Anesthesia and Intensive Care Department, Perugia University, 06121 Perugia, Italy; rachele.simonte@unipg.it (R.S.); edoardo.derobertis@unipg.it (E.D.R.); 2 Anesthesia and Intensive Care Unit, Department of Medical Surgical Science, University Hospital of Foggia, 71121 Foggia, Italy; antonella.cotoia@unifg.it; 3 Anesthesia and Intensive Care Unit, Department of Emergency, Health Integrated Agency of Friuli Centrale, Tolmezzo Hospital, 33028 Tolmezzo, Italy; luigi.vetrugno@asufc.sanita.fvg.it; 4 Internal Medicine and Nutritional Unit, ‘Madonna del Soccorso’ General Hospital, 63074 San Benedetto del Tronto, Italy; scarpidio@gmail.com; 5 Anesthesia and Intensive Care Unit, Azienda Ospedaliera Santa Maria of Terni, 05100 Terni, Italy; g.petroni@aospterni.it (G.M.P.); r.commissari@aospterni.it (R.C.); 6 Anesthesia and Neurointensive Care Unit, Department of Anesthesia and Intensive Care, Health Integrated Agency of Friuli Centrale, 33100 Udine, Italy; deana.cristian@gmail.com; 7 Anesthesia and Operating Room Department, Azienda Ospedaliera Santa Maria di Terni, 05100 Terni, Italy; r.monti@aospterni.it; 8 Department of Medicine and Surgery, University of Parma, 43126 Parma, Italy; elenagiovanna.bignami@unipr.it

**Keywords:** artificial intelligence, artificial nutrition, nutritional assessment

## Abstract

**Background**: Critical illness is characterized by profound and rapidly evolving metabolic derangements driven by systemic inflammation, hypercatabolism, fluid shifts, and endocrine dysregulation. These dynamic changes markedly limit the accuracy of predictive equations, increasing the risk of both underfeeding and overfeeding. Indirect Calorimetry Energy represents the gold standard for measuring energy expenditure, while bioelectrical impedance vector analysis (BIVA) provides complementary insights into hydration status, cellular integrity, and body cell mass. In palliative care, AI-supported integration of indirect calorimetry and BIVA enables goal-concordant artificial nutrition by aligning energy delivery with real-time metabolic status while minimizing symptom burden. Artificial intelligence (AI) has emerged as a promising tool to integrate these heterogeneous data streams and support adaptive nutritional strategies. **Methods**: We conducted a structured narrative review of the literature published between 2000 and 2025 using PubMed, Scopus, Embase, and Web of Science. Artificial intelligence was not used to perform the literature search or study selection. Instead, AI was analyzed as a clinical and technological component within the included studies and explored as a future enabling strategy. Eligible publications involved adult critically ill patients and addressed indirect calorimetry, BIVA-derived parameters, or AI-based metabolic modeling applied to nutritional support. Given the heterogeneity of study designs and outcomes, findings were synthesized qualitatively. **Results**: Predictive equations showed substantial inaccuracy in unstable metabolic states, with errors frequently exceeding ±20–40%. Indirect calorimetry enabled individualized assessment of energy expenditure but remained limited by intermittent availability. Serial BIVA assessments consistently identified clinically relevant alterations in hydration status, body cell mass, and phase angle, the latter being strongly associated with adverse outcomes. Studies incorporating AI demonstrated improved integration of calorimetry, BIVA, and clinical variables, allowing identification of metabolic phenotypes, anticipation of metabolic shifts, and generation of adaptive nutritional recommendations. **Conclusions**: This narrative review highlights the complementary roles of Indirect Calorimetry and BIVA in characterizing metabolic needs in critical illness. Artificial intelligence does not replace these tools but enhances their clinical utility by integrating multidimensional data into dynamic, patient-specific nutritional strategies. The combined AI–IC–BIVA approach represents a promising framework for metabolic precision nutrition in the ICU, warranting prospective validation.

## 1. Introduction

The metabolic response to critical illness is characterized by complex physiologic disturbances involving inflammation, hypercatabolism, altered substrate utilization, endocrine dysregulation, and significant variability in energy requirements [1]. These perturbations evolve rapidly and are influenced by numerous factors, including infection, shock, organ dysfunction, pharmacologic therapies, temperature fluctuations, and ventilator settings [1,2]. As a result, nutritional prescriptions based on static formulas provide inadequate coverage of true metabolic needs [1,2,3]. Underfeeding contributes to muscle wasting, impaired immune response, poor wound healing, and prolonged ventilator dependence, while overfeeding increases carbon dioxide production, exacerbates respiratory workload, fosters hyperglycemia, and promotes hepatic steatosis [4,5]. In this context, precise assessment of energy expenditure and body composition is essential for tailoring nutritional support to the individual patient [6].

Indirect calorimetry, directly measures energy expenditure and Q/R ratio and provides physiologically grounded insight into metabolic activity [7]. Despite its advantages, calorimetry is often used intermittently, limiting its ability to track rapid metabolic fluctuations [8]. Bioelectrical impedance vector analysis offers complementary information regarding hydration, cellular integrity, and inflammatory status [9,10]. Artificial intelligence enhances the clinical utility of these tools by integrating heterogeneous physiologic inputs into dynamic predictive models [11]. Through continuous learning, AI provides a more complete representation of metabolic demands and supports adaptive nutritional adjustment [12]. The integration of these modalities establishes the foundation for a precision-oriented approach to nutritional support [13].

## 2. Methods

This structured narrative review was developed to synthesize current evidence on metabolic monitoring, indirect Calorimetry, bioelectrical impedance vector analysis BIVA and artificial intelligence as applied to nutritional management in critical illness.

A comprehensive search strategy was constructed using MEDLINE, Embase, and Web of Science from January 2000 to December 2025. Search terms included combinations of “indirect calorimetry,” “bioelectrical impedance vector analysis,” “critical illness,” “energy expenditure,” “metabolic monitoring,” “artificial intelligence,” “machine learning,” “nutrition support,” and “ICU metabolism.” The search incorporated both MeSH terms and free-text keywords to maximize retrieval sensitivity. Reference lists of key articles were screened to identify additional relevant publications. Studies were included if they involved adult critically ill patients and examined at least one of the following: energy expenditure measurement, BIVA-derived body composition insights, AI-based metabolic prediction, or clinical integration of these modalities into nutritional therapy. We excluded pediatric studies, case reports, non-ICU populations, and articles lacking metabolic or nutritional relevance.

Extracted variables included study design, patient population, monitoring methodology, physiological parameters assessed, primary metabolic findings, and relevance to an integrated metabolic model. Due to heterogeneity of study protocols, measurement techniques, and outcome definitions, quantitative pooling was not feasible. Instead, we performed a qualitative synthesis centered on identifying recurring mechanistic principles and integrating them into a cohesive conceptual framework. In parallel, we examined barriers to implementation and explored potential avenues for future research required to validate an AI-driven metabolic precision strategy in critical care.

This review was conducted using a structured, transparent methodology appropriate to a structured narrative review. This manuscript is a narrative review, not a systematic review; strict adherence to PRISMA is therefore not required. However, in the interest of transparency, we applied elements of the PRISMA checklist where applicable and documented deviations with explicit justification (see Supplementary Table S1). Although a formal PRISMA flow diagram was not constructed, the selection process followed a structured, two-reviewer approach. The goal was to ensure comprehensive and transparent coverage of the literature related to indirect calorimetry, bioelectrical impedance vector analysis, and artificial intelligence applied to nutritional support in critical illness [14,15].

## 3. Search Strategy

A comprehensive literature search was performed across MEDLINE (via PubMed), Embase, and Web of Science, covering the period from January 2000 to December 2024. The search combined controlled vocabulary terms (MeSH and Emtree) with free-text keywords to maximize sensitivity. Search terms included:

- “indirect calorimetry” OR “energy expenditure”;
- “bioelectrical impedance vector analysis” OR “BIVA”;
- “critical illness” OR “intensive care unit”;
- “artificial intelligence” OR “machine learning” OR “predictive modeling”;
- “nutrition support” OR “clinical nutrition”.

Boolean operators and proximity filters were applied to refine results. No language restrictions were imposed initially; however, only English-language publications were retained during screening.

## 4. Eligibility Criteria

Studies were considered eligible if they met the following criteria:

1. Population: adult critically ill patients (ICU or high-dependency units).
2. Intervention/Exposure: use of indirect calorimetry, BIVA, AI-based prediction models, or integrated metabolic monitoring systems.
3. Outcomes: metabolic parameters (energy expenditure, body composition, hydration status), nutritional adequacy, predictive accuracy, or clinical outcomes related to metabolism.
4. Study Design: randomized trials, observational studies, cohort analyses, cross-sectional studies, technological validation studies, and methodological papers describing AI models.

The following were excluded:

- Pediatric studies;
- Case reports or case series with n < 10;
- Non-human studies;
- Articles without relevance to metabolic or nutritional monitoring;
- Purely engineering/algorithm papers with no clinical application.

**Study Selection**

Titles and abstracts were first screened for relevance by two independent reviewers. Full texts were subsequently assessed for compliance with eligibility criteria. Disagreements were resolved through discussion and consensus. Although a formal PRISMA flow diagram was not constructed, the selection process followed a structured approach similar to systematic reviews (Table 1).

## 5. Data Extraction and Synthesis

Data extracted from the included studies encompassed:

- Study design and sample characteristics;
- Type and protocol of indirect calorimetry;
- BIVA methodology, vector interpretation, and frequency of measurement;
- AI model architecture, input variables, training/validation strategy;
- Metabolic outcomes (e.g., energy expenditure, body cell mass);
- Clinical endpoints (e.g., mortality, ventilator duration).

Because of heterogeneity in measurement techniques, device characteristics, AI model construction, and outcome definitions, quantitative synthesis was not feasible. Instead, a qualitative, narrative synthesis approach was adopted, prioritizing recurring physiologic mechanisms, converging trends, and translational implications.

## 6. Risk of Bias and Quality Considerations

Although formal risk-of-bias tools typically used in systematic reviews (e.g., Cochrane RoB 2, ROBINS-I, GRADE) were not applied—and no quality threshold was imposed for study exclusion, given the heterogeneity of study designs and the nascent nature of the AI-in-ICU-nutrition field—studies were qualitatively evaluated across the following dimensions:

- Methodological clarity;
- Appropriateness of measurement protocols;
- Completeness of reporting;
- Clinical relevance and applicability to the ICU setting. Studies with major methodological concerns (e.g., unclear measurement conditions, absence of patient demographics, no validation of AI models) were noted as limitations and interpreted with greater caution in the synthesis.

## 7. Indirect Calorimetry in Critical Illness

Indirect calorimetry is the most accurate available method to assess energy expenditure in critically ill patients. It quantifies oxygen consumption and carbon dioxide production, allowing calculation of metabolic rate using established stoichiometric principles [21]. Calorimetry reflects the interplay of pathophysiological factors such as systemic inflammation, neuroendocrine activation, muscle activity, thermogenesis, and ventilator settings. Numerous studies have shown that predictive equations such as Harris–Benedict, Penn State, and Ireton–Jones frequently fail to estimate energy requirements accurately in the ICU. These discrepancies are amplified in patients with complex conditions such as sepsis, trauma, burns, ARDS, and multi-organ dysfunction, where metabolic changes occur rapidly and unpredictably [22].

Clinical consequences of inadequate estimation include unintended hypocaloric or hypercaloric feeding. Underfeeding promotes negative nitrogen balance, immune dysfunction, muscle wasting, and delayed recovery. Overfeeding increases carbon dioxide production, complicates ventilator weaning, causes hyperglycemia, and increases hepatic fat deposition. In such settings, indirect calorimetry provides essential guidance for defining appropriate caloric targets [23].

Despite its value, Indirect Calorimetry, continuous calorimetry remains uncommon due to cost and logistical constraints.

## 8. The Clinical Relevance of the Q/R Ratio Measured by Indirect Calorimetry and Its Role in Guiding Nutritional Therapy

In the context of precision nutrition for critically ill patients, the Q/R ratio has been proposed as a clinically relevant index for aligning nutritional delivery with the patient’s measured metabolic demand [24]. When derived from indirect calorimetry, this ratio may support a shift from population-based caloric estimates toward individually adjusted nutritional prescriptions [23].

## 9. Understanding the Q/R Ratio

- Q: The energy expenditure measured by indirect calorimetry (kcal/day), capturing the patient’s instantaneous metabolic workload.
- R: The caloric intake delivered through enteral or parenteral nutrition.

The Q/R ratio expresses the alignment between what the patient actually needs and what the ICU team is providing. Its value offers actionable insights into the risk of underfeeding, overfeeding, or optimal metabolic matching [23,24].

Q/R Ratio Interpretation Thresholds

**Figure 1:**
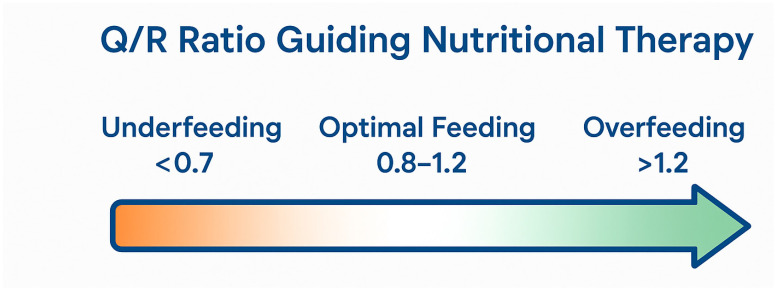
Respiratory Quotient (RQ) values as indicators of nutritional status.

Q/R < 0.8—Risk of Underfeeding
  When intake significantly lags behind measured needs, the patient remains exposed to persistent catabolism, impaired wound healing, immunosuppression, and delayed recovery. Early identification through indirect calorimetry allows for a targeted escalation of caloric and protein delivery, mitigating metabolic debt.
2. Q/R 0.8–1.0—Optimal Alignment
  This range reflects a metabolically synchronized feeding strategy, where the provided nutrition supports structural recovery, immune competence, and functional rehabilitation without overwhelming the patient’s capacity to metabolize substrates.
3. Q/R > 1.0—Risk of Overfeeding
  Excessive caloric delivery—often unrecognized in traditional approaches—exposes the critically ill patient to hypercapnia, hepatic steatosis, glycemic instability, and increased ventilatory burden. Monitoring Q/R enables timely de-escalation, preventing iatrogenic metabolic stress (Figure 1).

## 10. Why Q/R Matters in Critical Care

Indirect calorimetry offers a real-time, high-fidelity reflection of the patient’s metabolic status, capturing dynamic shifts driven by inflammation, sedation, fever, mechanical ventilation, and evolving organ dysfunction [23,24,25,26]. Through the Q/R lens, nutritional therapy evolves from a static intervention into an adaptive strategy aligned with disease trajectory.

The use of Q/R monitoring may offer several clinically relevant applications:

- Precision in nutritional titration, especially in complex or unstable metabolic states.
- Early identification of metabolic intolerance, allowing modulation of substrate composition (e.g., carb-to-fat ratio, protein prioritization).
- Risk mitigation for refeeding syndrome, through controlled, monitored advancement.
- Support for clinical decision-making, including timing of mobilization, fluid management, and ventilatory strategies.
- Enhanced outcome prediction, as persistent Q/R mismatch correlates with adverse trajectories.

Implementation of Q/R Monitoring in ICU Practice

Embedding Q/R within ICU workflows requires a structured, multidisciplinary approach—critical care physicians, dietitians, nurses, and metabolic experts collaborating on a shared data framework [27]. When applied systematically, Q/R monitoring may support structured clinical decisions including:

- Daily nutrition rounds,
- Escalation/de-escalation algorithms,
- Metabolic risk stratification,
- Documentation and accountability for nutritional adequacy.

Future Directions for Q/R Integration

As individualized approaches to ICU nutrition gain increasing clinical attention, the Q/R ratio provides a clinically accessible metric for evaluating the alignment between measured energy expenditure and nutritional delivery [26,28]. By supporting continuous adjustment of artificial nutrition to the patient’s actual metabolic state, clinicians may reduce the risk of misfeeding and improve nutritional precision.

Artificial intelligence offers a mechanism to integrate Indirect Calorimetry measurements with dynamic physiologic data streams, producing semi-continuous estimates of metabolic rate. Such models may compensate for measurement gaps, enabling more accurate and timely adjustments to nutritional prescriptions [29]. When combined with serial BIVA assessments, calorimetry-derived insights become part of a broader picture of metabolic status, supporting a precision-oriented nutritional strategy [30].

## 11. Role of Bioelectrical Impedance Vector Analysis

Bioelectrical impedance vector analysis has emerged as a valuable tool for assessing hydration status, cellular integrity, and body composition in critically ill patients. Unlike conventional bioimpedance methods that estimate absolute fluid volumes, BIVA evaluates the vector relationship between resistance and reactance, providing a qualitative and quantitative assessment of tissue hydration and membrane function [31]. In the context of critical illness, where inflammation, capillary leak, and aggressive fluid resuscitation frequently alter extracellular and intracellular compartments, BIVA offers a dynamic window into physiologic derangements [32]. Changes in vector position reflect shifts in tissue hydration, reductions in body cell mass, and alterations in cellular health driven by catabolic processes [33].

Fluid overload is common in critically ill patients and is strongly associated with adverse outcomes including respiratory failure, impaired oxygenation, prolonged mechanical ventilation, decreased renal recovery, and increased mortality. Traditional clinical tools such as physical examination, central venous pressure, and cumulative fluid balance are often insensitive or misleading, particularly in the presence of hypoalbuminemia or altered vascular permeability. BIVA identifies early fluid shifts by detecting reductions in resistance and alterations in vector trajectory, offering clinicians a more nuanced understanding of hydration status [34]. This information has significant implications for nutritional therapy, as fluid overload can impair gastrointestinal absorption, increase metabolic inefficiency, and promote tissue edema that compromises nutrient delivery.

Beyond hydration, BIVA provides insight into protein-energy status through the evaluation of body cell mass. Declines in reactance reflect loss of cellular integrity and reduced cell mass, hallmarks of hypercatabolism. These patterns may reveal inadequate protein intake, ongoing inflammation, or critical illness myopathy. Because loss of cell mass is strongly linked to impaired functional recovery and increased mortality, early detection is essential. Serial BIVA assessments enable tracking of metabolic recovery or deterioration, complementing calorimetry-derived energy measurements and guiding adjustments to protein dosing.

Furthermore, BIVA offers advantages related to its non-invasive nature, easy of repetition, and ability to provide immediate results. Serial measurements can be obtained even in hemodynamically unstable or mechanically ventilated patients, enabling time-series analysis that supports integration with artificial intelligence platforms. This allows BIVA to serve as a continuous biomarker of metabolic resilience and recovery potential.

Among BIVA derived parameters, the phase angle (PhA) stands out as a powerful, independent predictor of clinical outcomes.

Phase angle (PhA) is derived directly from the raw electrical measurements obtained during bioelectrical impedance analysis. The device applies a small alternating current through the body and measures two key components:

- Resistance (R): the opposition offered by body fluids to the electrical current.
- Reactance (Xc): the delay caused by cell membranes, which act as capacitors.

These two vectors form the basis of the phase shift between voltage and current.

The phase angle is simply the arctangent of the ratio between reactance and resistance.

## 12. The Formula

The phase angle (PhA) is a bioelectrical impedance parameter that reflects cell membrane integrity and functionality, converted from radiants to degrees.

Because critically ill patients often exhibit membrane dysfunction, fluid shifts, and reduced body cell mass, the Xc/ratio declines, generating a lower phase angle, which is why PhA is so tightly linked to prognosis. It is not influenced by regression equations or predictive models. It reflects pure, direct bioelectrical properties of tissues. It is, therefore objective, reproducible, and independent of operator interpretation. This makes phase angle a robust metric for tracking cellular health, nutritional status, and metabolic recovery in real time.

Phase Angle (°) = arctan(Xc/R) × (180\pi)

## 13. What This Means Operationally

- Higher Xc → stronger capacitive behavior → healthier, more intact cell membranes → higher PhA.
- Higher R → more opposition due to fluid depletion → lower PhA if not accompanied by proportional Xc.

Because critically ill patients often exhibit membrane dysfunction, fluid shifts, and reduced body cell mass, the Xc/R ratio declines, generating a lower phase angle, which is why PhA is so tightly linked to prognosis.

## 14. Why This Calculation Matters Clinically

- It is not influenced by regression equations or predictive models.
- It reflects pure, direct bioelectrical properties of tissues.
- It is therefore objective, reproducible, and independent of operator interpretation.

This makes phase angle a robust metric for tracking cellular health, nutritional status, and metabolic recovery in real time.

## 15. Phase Angle as a Biological Performance Indicator

The phase angle reflects the integrity and functionality of cell membranes and the proportion of metabolically active body cell mass. A low PhA is consistently associated with impaired cellular health and reduced anabolic capacity—two elements deeply intertwined with morbidity in critical illness. Evidence shows strong correlations between lower PhA values and:

- Prolonged ICU length of stay,
- Higher rates of infectious and non-infectious complications,
- Diminished response to artificial nutrition,
- Increased ventilator dependence,
- Greater risk of multi-organ dysfunction,
- Higher short- and long-term mortality.

Conversely, a preserved or improving PhA trajectory signals a patient with greater physiological reserve, more robust cell membrane function, and better capability to respond to metabolic stressors. Patients demonstrating PhA improvement during their ICU course typically show enhanced tolerance to nutritional interventions, a more efficient inflammatory resolution process, and faster functional recovery

Phase Angle Cut-off: Healthy vs. Critically Ill Patients

The phase angle (PhA) varies by age, sex, and body composition, but the literature consistently identifies clear thresholds distinguishing healthy individuals from critically ill patients.

## 16. Healthy Adults

- Typical values:
  Men: 5.5–7.5°;
  Women: 5.0–7.0°.
- Physiological cut-off for normality:
  >5.0–5.5° in most adults.
  These values indicate preserved cell membrane integrity and adequate body cell mass.

## 17. Critically Ill Patients

In critical illness, phase angle decreases markedly due to:

- Membrane dysfunction,
- Systemic inflammation,
- Loss of body cell mass,
- Impaired metabolic reserve.

The most accepted ICU cut-offs are:

- <4.0–4.5° → marker of high clinical risk
  (associated with increased mortality, prolonged ventilation, and poor nutritional responsiveness).
- <3.5° → strong independent predictor of adverse outcomes.
  Multiple studies identify this threshold as highly specific for ICU and in-hospital mortality.

Phase Angle Reference Values: Summary

**Figure 2:**
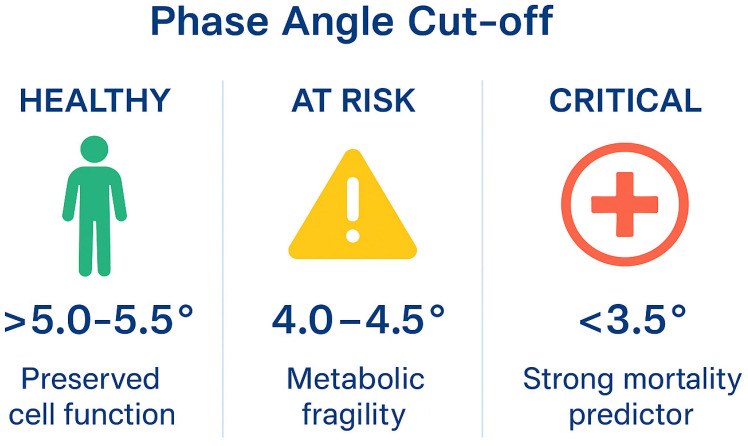
Phase angle Cut-Off Values for Clinical Risk Stratification.

- Healthy: PhA > 5.0–5.5°;
- Critically ill: PhA < 4.5° = high risk (Figure 2).

## 18. Implications for Nutritional Management

Integrating PhA into nutritional workflows allows the ICU team to shift from static, guideline-driven prescriptions to a precision-nutrition model grounded in dynamic biological data. Phase angle supports:

- Early identification of patients with severe metabolic vulnerability,
- Individualized titration of energy and protein targets,
- Real-time monitoring of nutritional responsiveness,
- Risk stratification for refeeding syndrome or catabolic drift,
- Alignment of nutrition therapy with the patient’s actual cellular performance rather than estimated needs alone.

The assessment of lean body mass is poised to become the real future target of nutritional therapy, particularly in guiding protein delivery. By shifting the focus from static weight-based formulas to direct evaluation of metabolically active tissue, clinicians will be able to tailor protein prescriptions with far greater precision—optimizing anabolism, preserving functional capacity, and ultimately improving clinical outcomes [35,36].

This approach ensures that artificial nutrition becomes not just a support measure but a strategic therapeutic lever, aligned with the patient’s evolving metabolic phenotype.

## 19. Operational and Outcome-Oriented Value

Embedding BIVA and PhA into ICU routines enhances clinical governance by enabling the team to:

- Prioritize resources toward high-risk patients,
- Objectively track metabolic recovery,
- Improve prognostic accuracy,
- Support early multidisciplinary discussions,
- Support transparent documentation and consistency in nutritional decision-making.

Serial BIVA-derived phase angle assessment may contribute to more individualized and outcome-oriented nutritional management in critically ill patients, pending prospective validation in adequately powered clinical trials.

## 20. Artificial Intelligence in Metabolic Monitoring

Artificial intelligence provides a powerful mechanism for synthesizing heterogeneous physiologic signals into comprehensive, continuously updated metabolic models. Machine-learning algorithms such as neural networks, random forest ensembles, gradient-boosting machines, and time-series prediction models can incorporate data from indirect calorimetry, BIVA, ventilator mechanics, hemodynamic measurements, inflammatory biomarkers, and laboratory studies. These systems excel at identifying nonlinear patterns, variable interactions, and temporal dynamics that escape traditional analytical approaches [37,38] (Table 2).

AI-based models demonstrate superior accuracy compared with conventional predictive equations. They can generate real-time or near-real-time estimates of Energy Expenditure, predict short-term changes in metabolic rate, and identify unique metabolic phenotypes including hypermetabolic, inflammatory, or hypo-metabolic states. These phenotypes have clinical relevance, as they correlate with outcomes, nutritional needs, and recovery trajectories [39].

In addition to prediction, AI enhances clinical decision-making by generating adaptive nutritional recommendations. Algorithms can detect early signs of metabolic deterioration, predict increasing catabolic drive, or identify fluid-overload states that may impede nutrient utilization. By integrating calorimetry and BIVA inputs, AI establishes a cohesive metabolic narrative that supports timely and individualized nutritional intervention. Ultimately, AI bridges the gap between intermittent measurement and continuous metabolic management [40].

## 21. The Integrated AI–IC–BIVA Framework

The integration of artificial intelligence, indirect calorimetry, and bioelectrical impedance vector analysis forms a unified framework for metabolic precision in the ICU. Each component contributes complementary insights: calorimetry quantifies real-time energy expenditure, BIVA characterizes hydration and cellular integrity, and AI contextualizes these measurements within the broader physiologic landscape. Together, they enable a dynamic, adaptive model of nutritional management capable of responding to rapid metabolic fluctuations [41]. This framework, which we refer to as the Metabolic Precision Loop, is predicated on continuous data flow, predictive analytics, and iterative refinement of nutritional targets.

The Metabolic Precision Loop operates through four interconnected phases. The first is the input phase, in which Indirect Calorimetry measurements, BIVA vectors, ventilator parameters, hemodynamic data, temperature profiles, laboratory markers, and clinical observations are gathered [42,43]. These inputs capture the metabolic, inflammatory, and fluid status of the critically ill patient. The second phase is the interpretation layer, in which artificial intelligence algorithms process incoming data to identify metabolic phenotype, detect early deterioration, and forecast energy expenditure. These models incorporate both static and dynamic variables, accommodating time-dependent interactions and nonlinear relationships. The third phase is the output phase, where AI-derived insights are translated into actionable clinical recommendations. These include caloric targets, protein dosing, feeding modality, timing adjustments, and fluid–nutrition alignment. The fourth phase is the feedback loop, in which repeated measurements are collected and compared to predicted trajectories. The model is refined continuously, ensuring that nutritional support remains aligned with evolving physiologic demands [41,44].

This closed-loop model offers substantial advantages over static approaches. It reduces reliance on fixed caloric prescriptions, intervenes earlier in episodes of metabolic instability, and provides clinically interpretable metrics of progress or deterioration. Importantly, such a framework promotes the alignment of nutritional delivery with cellular capacity for anabolic recovery, enhancing the potential for improvement in muscle mass, organ function, and long-term outcomes [45].

## 22. Comparative Evaluation: Traditional vs. Precision Approaches

Traditional nutritional strategies in the ICU rely heavily on predictive equations and clinical judgment. These methods are inherently limited by their inability to account for the rapid variability characteristic of critical illness. Predictive equations estimate caloric needs using demographic and anthropometric data, often without integrating ongoing physiologic changes such as fever, sedation, inflammatory storms, or ventilatory adjustments. As a result, they provide an oversimplified representation of metabolic demand, leading to inadvertent underfeeding or overfeeding [46].

In contrast, the AI–IC–BIVA precision model incorporates real-time physiologic data, enabling a dynamic, responsive approach. Instead of relying on fixed targets, clinicians receive continuously updated recommendations grounded in objective metabolic measurements. BIVA contributes critical information regarding cellular health and hydration that cannot be inferred from calorimetry alone. AI synthesizes these insights, translating them into predictive curves and actionable guidance [47].

The advantages of the precision model extend beyond accuracy. It supports earlier recognition of catabolic crises, enhances the capacity to tailor protein supplementation, and promotes more efficient weaning from mechanical ventilation through optimized energy balance. It also provides transparency and consistency in nutritional decision-making, reducing variation attributable to clinician experience or institutional practice. Ultimately, the AI–IC–BIVA model represents a fundamental shift from reactive to proactive nutritional therapy, aligning interventions with actual physiologic needs [45,48] (Table 3).

## 23. Operational Considerations and Implementation Challenges

Although the AI–IC–BIVA framework offers considerable potential, several operational challenges must be addressed before widespread clinical adoption can occur. One key challenge involves the variability of indirect calorimetry usage across institutions. Despite strong recommendations supporting its use, calorimetry remains inconsistently implemented due to concerns regarding device cost, maintenance requirements, staffing limitations, and the need for standardized measurement protocols. Variability in technique, timing, and interpretation can lead to inconsistent data quality, which in turn may limit the performance of AI algorithms that rely on reliable physiologic inputs [49,50].

Bioelectrical impedance vector analysis faces similar challenges, particularly related to operator experience, electrode placement, and device heterogeneity. Although BIVA is conceptually simple, accurate interpretation requires familiarity with vector trajectories, reference ellipses, and the physiologic significance of reactance and resistance changes. The absence of universal ICU-specific reference standards may complicate interpretation, especially in patients with extreme fluid overload or significant edema. Additionally, the timing of measurements relative to fluid administration, renal replacement therapy, or vasopressor adjustments can impact vector patterns. Ensuring consistency requires personnel training, clear protocols, and integration of BIVA measurement schedules into routine ICU workflows [51,52].

Artificial intelligence systems introduce an additional layer of complexity. Algorithm development requires large, high-quality datasets that reflect the diversity of ICU populations. The performance of AI models may be limited by biases in training data, insufficient representation of specific subgroups, or lack of interoperability among electronic medical record systems. Ethical concerns regarding data privacy, algorithm transparency, and the need for human interpretability must also be addressed. Furthermore, the implementation of AI-assisted decision support requires seamless integration with existing clinical pathways to avoid workflow disruptions or alert fatigue [53].

Technical considerations include the need for robust data pipelines that connect Indirect calorimetry, BIVA-analyzers, and bedside monitoring systems to centralized AI platforms. Real-time data acquisition and preprocessing are essential for generating timely predictions. Network reliability, cybersecurity protections, and fail-safe mechanisms must be established to maintain clinical safety. Additionally, clinicians must retain ultimate decision-making authority, with AI recommendations serving as supportive rather than prescriptive guidance [51,54].

## 24. Clinical Implications and Potential Benefits

The integration of AI, indirect calorimetry, and BIVA has the potential to significantly enhance the quality and precision of nutritional therapy in critically ill patients. By aligning caloric delivery with measured energy expenditure, the framework reduces the risk of misfeeding and its associated complications. Precise protein dosing informed by metabolic phenotype and cellular integrity may mitigate muscle wasting, promote anabolic recovery, and improve long-term functional outcomes. Early detection of fluid overload or dehydration through BIVA can refine fluid–nutrition interactions, ensuring that nutrient absorption and utilization are optimized [55,56].

AI-driven predictive modeling allows clinicians to anticipate metabolic shifts before they manifest clinically. This enables proactive adjustment of nutritional targets during periods of increased catabolic stress, such as sepsis, fever, or surgery, and ensures appropriate downscaling of caloric delivery during hypo-metabolic phases. The dynamic nature of the Metabolic Precision Loop supports continuous alignment between nutrition prescription and physiologic readiness, potentially reducing the incidence of feeding intolerance, hyperglycemia, and respiratory complications [57,58].

Beyond physiologic benefits, the framework may improve organizational efficiency by reducing practice variability and standardizing nutritional workflows. Objective, data-driven recommendations enhance consistency and provide a clearer rationale for nutritional decisions, facilitating interdisciplinary communication between physicians, dietitians, physiotherapists and nursing staff. Over time, this may contribute to improved resource utilization, shorter ICU stays, and better patient-centered outcomes [59].

## 25. Integration with Multidisciplinary ICU Practice

Successful implementation of the AI–IC–BIVA framework requires alignment with the multidisciplinary nature of ICU care [57]. Nutritional therapy intersects with numerous clinical domains, including mechanical ventilation, renal support, hemodynamic management, infection control, sedation practices, and rehabilitation strategies. Therefore, embedding precision nutrition within daily workflow necessitates collaboration across professional boundaries. Physicians, dietitians, nurses, respiratory therapists, pharmacists, and physiotherapists all contribute to metabolic homeostasis, and their coordinated actions influence nutritional efficacy. The Metabolic Precision Loop enhances this collaboration by providing a shared, objective representation of metabolic status. This fosters consistent decision-making and enables all team members to work toward unified therapeutic goals [57,60].

Dietitians play a central role as interpreters of metabolic information and facilitators of nutritional prescription. AI-driven caloric targets and BIVA-derived cell mass trends provide them with continuous insight into the adequacy of nutritional plans. Nurses contribute crucial observational data regarding feeding tolerance, gastrointestinal motility, glycemic control, and fluid balance, all of which influence metabolic performance. Respiratory therapists offer valuable input by monitoring ventilator demands and evaluating the influence of nutritional changes on respiratory workload [57,58]. Pharmacists assist with the optimization of micronutrient supplementation and glycemic management, while physiotherapists provide feedback on functional trajectory and muscle preservation. Such multidisciplinary engagement ensures that nutritional interventions are integrated into a broader therapeutic strategy tailored to each patient’s evolving clinical course [58,61].

## 26. Palliative Care, Artificial Nutrition, and AI-Enhanced Monitoring (IC and BIVA)

In the palliative care setting, nutritional management plays a strategic role in sustaining functional capacity, mitigating symptom burden, and preserving patient dignity throughout the disease trajectory. Even when curative options are no longer feasible, maintaining adequate protein–energy intake contributes to reducing infection risk, attenuating loss of lean body mass, and supporting the patient’s ability to engage in basic activities of daily living. Evidence shows that malnutrition and sarcopenia are independently associated with increased mortality, higher symptom intensity, reduced tolerance to therapies, and greater caregiver burden. Within this context, artificial nutrition—when aligned with patient-centered goals—remains a core component of supportive care [62].

The integration of indirect calorimetry (IC) and bioelectrical impedance vector analysis (BIVA) enhances clinical decision-making by enabling individualized, minimally burdensome monitoring of metabolic shifts that frequently characterize end-of-life physiology. IC provides real-time assessment of resting energy expenditure, preventing both underfeeding and overfeeding—conditions that may exacerbate fatigue, dyspnea, or fluid overload in frail patients. In parallel, BIVA offers dynamic insight into hydration patterns, inflammatory edema, and cellular integrity, supporting tailored interventions that can stabilize body cell mass and reduce the risk of pressure injuries or mobility decline [63,64].

Artificial intelligence can unify these data streams into adaptive algorithms capable of predicting catabolic acceleration, infection risk, or impending functional decline. This approach fosters more precise titration of enteral or parenteral nutrition, aligning treatment intensity with patient preferences and avoiding futile interventions. By anticipating metabolic deterioration and optimizing nutrient assimilation, AI-supported IC and BIVA workflows have the potential to improve quality of life and reduce unplanned hospitalizations—key performance indicators in contemporary palliative medicine [65,66].

Overall, a precision-nutrition paradigm grounded in AI-enhanced monitoring supports a more ethical, personalized, and function-preserving approach to palliative care, ensuring that nutritional therapy remains proportionate, goal-concordant, and integrated within the broader multidisciplinary framework [67].

## 27. Ethical, Regulatory, and Governance Considerations

The integration of artificial intelligence into clinical decision-making raises important ethical and regulatory considerations. Transparency is essential to ensure that clinicians understand how AI-generated recommendations are formulated. Models must be interpretable, with clear descriptions of input variables, algorithmic structure, performance metrics, and limitations. Black-box models that provide recommendations without explanatory context may undermine trust, particularly in high-stakes decision environments such as the ICU [68].

Data governance is another critical area. AI systems rely on continuous acquisition of patient data, necessitating strict adherence to privacy regulations and robust cybersecurity protections. Institutions must ensure secure data storage, encrypted transfer protocols, and appropriate consent processes where applicable. Additionally, developers must guard against algorithmic bias, ensuring that models perform equitably across demographic and clinical subgroups [69]. Bias may arise from imbalanced training datasets, incomplete data capture, or structural inequities in healthcare systems. Rigorous validation across multiple centers and diverse patient populations is required to ensure fairness and generalizability [70,71].

Clear guidelines must govern the responsibilities of clinicians, developers, and institutions. AI should augment—not replace—clinical judgment [72]. Clinicians must remain accountable for interpreting AI-derived insights within the context of each patient’s unique clinical situation. Instituting governance frameworks that encompass auditing, performance monitoring, and continual model updating will be essential to maintain patient safety and ensure sustained model accuracy [73].

### 27.1. Educational Requirements and Cultural Adaptation

The successful deployment of this framework depends on clinician education and cultural adaptation. Training programs must familiarize staff with the principles of indirect calorimetry, BIVA interpretation, and AI-based decision support [16,17]. Education should emphasize the strengths and limitations of each modality, highlighting scenarios where integration is most beneficial. Cultivating a culture of technological openness and data-driven decision-making will enhance adoption. Interdisciplinary workshops, bedside teaching, and simulation-based training may facilitate knowledge dissemination and ensure consistent practice [18,19,20].

### 27.2. Future Research Priorities

It must be acknowledged that the Metabolic Precision Loop, as described in this review, remains a conceptual framework derived from convergent indirect evidence and pathophysiological reasoning. Its clinical validity and impact on patient-centered outcomes—including mortality, ventilator-free days, functional recovery, and quality of life—must be prospectively tested in well-designed randomized controlled trials before it can be recommended for routine clinical implementation. The current review is intended to provide the theoretical and evidentiary foundation for such trials, not to substitute for them.

The integration of artificial intelligence, indirect calorimetry, and BIVA into a unified precision nutrition framework opens several avenues for future investigation. First, randomized controlled trials are required to evaluate whether AI-guided nutritional strategies improve clinical outcomes compared with standard care. Such trials should assess not only traditional endpoints, including mortality, length of stay, and ventilator-free days, but also patient-centered outcomes such as functional recovery, muscle strength, quality of life, and long-term independence. Because nutritional interventions influence both acute physiology and post-intensive care trajectories, measuring outcomes that extend beyond hospital discharge is essential. Trials should incorporate repeated BIVA measurements and calorimetry-based adjustments to validate the real-time benefits of the Metabolic Precision Loop.

Further development of continuous or near-continuous calorimetry technologies would enhance the fidelity of metabolic monitoring. Current calorimetry devices provide intermittent data, leaving gaps during periods of physiologic instability. Emerging techniques capable of providing continuous VO_2_ and VCO_2_ measurements would allow AI models to capture metabolic fluctuations with greater temporal resolution. Integration of continuous calorimetry into existing ventilator platforms or standalone devices could significantly enhance the responsiveness of nutritional therapy.

The role of molecular and biochemical biomarkers in metabolic phenotyping warrants deeper exploration. Metabolomics, proteomics, lipidomics, and inflammatory mediator profiling may reveal distinct metabolic signatures associated with hypercatabolism, immunosuppression, anabolic resistance, or maladaptive inflammation. Incorporating these biomarkers into AI models could refine phenotype classification and improve prediction accuracy. Similarly, the integration of imaging-based techniques, such as ultrasound for muscle thickness or echogenicity assessment, may provide additional context regarding muscle mass preservation and nutritional adequacy.

The expansion of high-quality, ICU-specific BIVA reference standards is needed. Current reference ellipses are primarily derived from healthy populations and may not accurately represent the physiological states of critically ill patients. Large-scale data collection initiatives could generate BIVA reference curves tailored to various ICU subgroups, including sepsis, trauma, burns, neurological injury, and cardiac surgery. This would enhance interpretability and improve the clinical relevance of vector analysis.

The application of reinforcement learning and advanced time-series models represents a promising frontier. These algorithms can adapt nutritional recommendations based on observed patient responses, learning the most effective strategies over time. Such models could optimize the timing and composition of nutritional interventions, balancing caloric delivery, protein provision, and fluid constraints in a tailored manner. Importantly, reinforcement learning systems require stringent safety controls to prevent harmful recommendations, making collaboration between clinicians and data scientists crucial.

Finally, research examining organizational and economic implications is essential. Precision nutrition may require investment in calorimetry devices, BIVA equipment, data infrastructure, and AI platforms. Analyses should quantify cost savings associated with reduced complications, shorter ICU stays, improved functional recovery, and decreased readmission rates. Demonstrating economic value may accelerate adoption across healthcare systems and support long-term sustainability.

A key question for future research is: who can benefit from the conclusions of this narrative review, and how? Intensive care physicians, clinical dietitians, and nursing staff represent the primary clinical stakeholders, as this framework directly informs bedside nutritional decision-making. Biomedical engineers and data scientists may benefit by identifying the specific input variables and algorithmic requirements needed to develop validated AI models for ICU nutrition. Hospital administrators and health system planners can use these findings to guide investment in metabolic monitoring infrastructure. Researchers in clinical nutrition and critical care may find this synthesis useful as a foundation for designing prospective trials. Finally, in the palliative care setting, interdisciplinary teams caring for patients with advanced illness can apply the IC-BIVA-AI framework to align nutritional interventions with individual metabolic trajectories and patient-centered goals, minimizing symptom burden while preserving functional capacity. Translating these findings into practice will require context-specific implementation strategies tailored to each of these stakeholder groups.

### 27.3. Strengths and Limitations of the Proposed Framework

The AI–IC–BIVA framework offers several strengths. It integrates complementary modalities to provide a holistic understanding of metabolic status, aligns nutritional therapy with objective physiologic data, and enables proactive rather than reactive intervention. Through iterative refinement, the model enhances accuracy and responsiveness. However, limitations include dependence on device availability, need for robust data infrastructure, and potential challenges in clinician adoption. Ensuring equitable access across institutions and avoiding digital disparities are important considerations.

### 27.4. Barriers, Limitations, and Practical Considerations

Despite the strong rationale, implementation challenges remain. IC and BIVA availability varies across ICUs, and measurement protocols require training and workflow integration.

From a methodological standpoint, this review is also limited by the selection of databases used. The search was conducted across MEDLINE, Embase, and Web of Science; however, grey literature sources—including conference proceedings, preprints, institutional reports, and non-indexed clinical protocols—were not systematically searched and may contain relevant data not captured in this synthesis. Additionally, only English-language publications were retained, which may have introduced language bias. These limitations should be considered when interpreting the breadth of the evidence base presented.

AI models require high-quality datasets, careful validation, explainability, and robust governance to avoid bias and ensure clinical trust.

Ethical and operational frameworks must ensure that AI recommendations complement rather than dominate clinical reasoning.

Furthermore, prospective trials are needed to determine whether AI-guided metabolic strategies can improve hard outcomes such as mortality, ventilator dependence, renal failure, and long-term functional recovery. Early signals are promising, but evidence must mature through multicenter studies and real-world implementation pilots.

## 28. Conclusions

Critical illness is characterized by extreme metabolic heterogeneity, driven by inflammation, hypercatabolism, fluid redistribution, endocrine dysregulation, and rapidly changing organ support requirements. In this context, traditional nutrition strategies based on predictive equations and static prescriptions are intrinsically inadequate and frequently result in clinically relevant underfeeding or overfeeding. This narrative review highlights how objective metabolic monitoring tools—indirect calorimetry and bioelectrical impedance vector analysis—provide complementary and physiologically grounded insights that are essential for aligning nutritional therapy with the patient’s true metabolic state.

Conditions that critically influence nutritional effectiveness and clinical outcomes: By shifting attention from body weight-based targets to biologically meaningful markers of cellular performance, BIVA supports a more nuanced and outcome-oriented approach to nutritional management.

The resulting AI–IC–BIVA framework, conceptualized as a Metabolic Precision Loop, represents a promising conceptual framework oriented toward more proactive and individualized nutritional therapy, warranting prospective validation in multicenter randomized controlled trials before it can be adopted as a clinical standard. By continuously aligning energy and protein delivery with the patient’s evolving metabolic and cellular capacity, this approach has the potential to mitigate iatrogenic metabolic stress, preserve lean body mass, facilitate ventilator weaning, and support functional recovery. Beyond individual patient benefits, the framework also promotes standardization, transparency, and consistency in nutritional decision-making, enhancing interdisciplinary communication and clinical governance within the ICU.

Nevertheless, several challenges must be addressed before widespread implementation. These include variability in availability and standardization of calorimetry and BIVA measurements, the need for robust data infrastructure, clinician training in interpretation of advanced metabolic metrics, and the establishment of ethical and regulatory frameworks to ensure transparency, explainability, and accountability of AI-assisted decision support. Artificial intelligence must remain a supportive tool, embedded within multidisciplinary workflows and governed by clear clinical oversight.

Future research should focus on prospective, multicenter trials to evaluate whether AI-guided precision nutrition translates into meaningful improvements in clinical outcomes, functional recovery, and long-term quality of life. Additional efforts are required to develop ICU-specific BIVA reference standards, integrate continuous or near-continuous calorimetry technologies, and explore advanced AI methodologies such as reinforcement learning under strict safety constraints. Economic and organizational evaluations will also be essential to determine the sustainability and scalability of this precision nutrition model.

In the palliative care setting, the integration of indirect calorimetry, bioelectrical impedance vector analysis, and artificial intelligence enables a precision-nutrition approach that supports functional capacity, mitigates symptom burden, and preserves patient dignity by aligning artificial nutrition with individualized metabolic trajectories, patient-centered goals, and ethically proportionate care.

**Figure 3:**
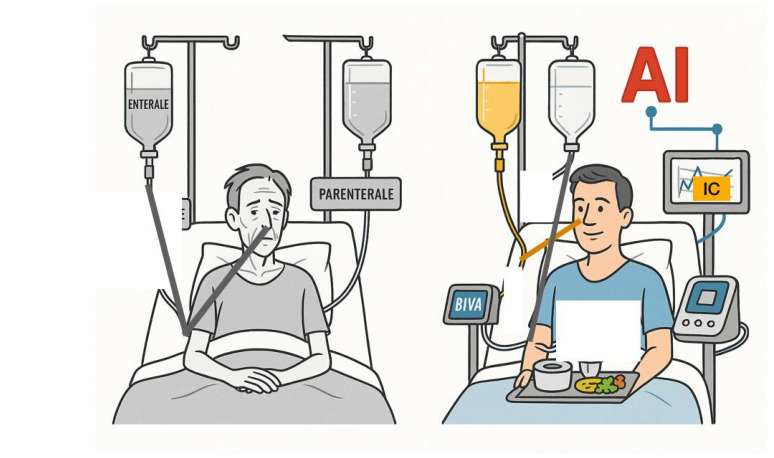
Effect of AI-IC-BIVAN combination on patients.

In conclusion, the integration of Indirect Calorimetry, BIVA- and artificial intelligence offers a coherent and physiologically grounded pathway toward metabolic precision nutrition in critical care. By reframing artificial nutrition as a dynamic, data-driven therapeutic strategy rather than a static supportive measure; this framework has the potential to redefine best practices and elevate the role of nutrition as a central pillar of personalized critical care medicine (Figure 3).

## Figures and Tables

**Table 1:** Characteristic of main included studies.

References	Setting/Population	Tool	Key Findings	Clinical Implications	DOI/PMID	Sample Size	Design
[15]	ICU ventilated	BIVA	Vector (RXc) & PA predict mortality	Supports fluid-sensitive nutrition	https://doi.org/10.1097/MCC.0000000000000840	152	Prospective
[16]	Sepsis/shock	Indirect Calorimetry	REE differs 20–30% from equations	IC prevents under/overfeeding	https://doi.org/10.1038/s41430-021-01012-2	124	Multicenter
[17]	ICU COVID-19	IC	IC changed prescription in ~90%	Supports routine IC use	https://doi.org/10.1016/j.clnu.2024.07.048	432	Prospective
[18]	Mixed ICU	BIA/PA	PA predicts mortality & ICU weakness	PA as metabolic severity marker	https://doi.org/10.1186/s13054-021-03830-z	93	Prospective
[19]	Renal/fluid overload	BIVA	Introduced RXc graph; vector predicts hydration	Foundation of BIVA methodology	https://doi.org/10.1038/ki.1994.305	N/A	Methodological
[20]	Renal patients	BIVA	Overload vector displacement patterns	Defines clinical interpretation	PMID: 8676831	N/A	Clinical validation
[9]	Hospitalized/chronic disease	Phase Angle	Low PA predicts mortality & complications	Robust prognostic marker	https://doi.org/10.1016/j.clnu.2012.05.008	N/A	Clinical study

**Table 2:** Metabolic Biomarkers in Critical Illness (IC, BIVA, PA, ECW/ICW, Inflammation, Catabolism).

Biomarker	Source/Tool	Physiological Meaning	Clinical Interpretation in ICU	Key Cut-Offs/Thresholds	Implications for Nutrition Therapy
Resting Energy Expenditure (REE)	Indirect Calorimetry	Real metabolic rate (O_2_ consumption + CO_2_‚ production)	Hypermetabolism, metabolic stress, under/overfeeding risk	>110–120% predicted = hypermetabolic	Guides caloric prescription; prevents misfeeding
RQ (Respiratory Quotient)	Indirect Calorimetry	Substrate oxidation (carbs vs. fats)	RQ > 1.0: overfeeding; RQ < 0.7: lipolysis/ketosis	RQ 0.85–0.95 optimal	Fine-tunes macronutrients (CHO/lipids)
Phase Angle (PA)	BIA/BIVA	Cell membrane integrity & capacitance	Low PA: inflammation, catabolism, poor prognosis	PA ≤ 4 Â early ICU = high risk	Adjust protein strategy; monitor catabolism
Vector Position (RXc)	BIVA	Hydration + cellularity	Right-shift: ECW expansion; Up-shift: high cell mass	RXc ellipses (50/75/95%)	Determines fluid-tolerant vs. fluid-sensitive nutrition
Vector Length	BIVA	Soft-tissue mass & ICW content	Short vector: edema/ECW; Long vector: preserved cell mass	Trajectory more important than single value	Tailors protein targets; identifies sarcopenia
ECW/ICW Ratio	BIA/BIVA	Hydration & cellular distribution	ECW/ICW = edema, inflammation, catabolic muscle loss	>1.0–1.2 high risk	Adjust EN volume; reduce overload; manage FO
TBW/Predicted TBW	BIA	Global hydration	Underestimation by fluid balance charts	TBW with stable weight = hidden FO	Guides EN rate, fluid restriction
Urinary Nitrogen Loss	Catabolic marker	Protein catabolism rate	BUN: hypercatabolic state requiring protein escalation	>15–20 g/d = high catabolism	Adjust protein 1.5–2.2 g/kg
CRP/IL-6	Inflammatory biomarkers	Inflammatory burden	High inflammation altered substrate utilization	CRP > Severe stress	IC-guided conservative energy in hyperinflammation
Lactate	Global perfusion marker	Mismatch between supply/demand	Persistent lactate metabolic stress	>2–4 mmol/L	Delay full feeding; avoid overfeeding
Glucose Variability	Metabolic instability	Stress response, insulin resistance	variability correlates with mortality	CV > 20%	Stabilize feeding; glucose-adaptive algorithms
SOFA/Organ Support Load	Clinical severity	Multiorgan impact on metabolism	Higher organ support higher metabolic volatility	SOFA >10	Needs frequent IC; cautious EN advancement
Muscle Ultrasound + PA	Sarcopenia markers	Structural + electrical muscle integrity	Loss of cross-section + PA drop = severe muscle catabolism	>10% decrease in 7 days	Increase protein; consider PN supplementation

**Table 3:** Metabolic and Nutritional Variables Assessed With and Without AI Support.

Domain	Variable	Measured Without AI	AI-Enhanced Interpretation
Energy metabolism	Resting Energy Expenditure (REE)	Indirect calorimetry (IC)	Prediction between IC measurements; trend forecasting
Substrate utilization	Respiratory Quotient (RQ)	IC-derived	Early detection of over/underfeeding patterns
Energy delivery	Q/R ratio	Manual calculation	Automated alerts and adaptive targets
Hydration status	RXc vector position	BIVA-Akern	Pattern recognition of fluid-sensitive phenotypes
Cellular health	Phase Angle (PhA)	BIVA-Akern	Prognostic stratification; trajectory analysis
Body composition	Body Cell Mass trends	Serial BIVA	Prediction of catabolic drift
Protein needs	Nitrogen balance proxies	Conventional methods	AI-driven protein titration
Metabolic phenotype	Hyper/hypometabolism	X	AI-based clustering
Nutritional risk	Under/overfeeding risk	Retrospective	Real-time predictive alerts
Decision support	Nutrition prescription	X	Dynamic, patient-specific recommendations

## Data Availability

Data sharing is not applicable to this article as no new data were created or analyzed in this study. This is a narrative review based on previously published studies.

## Supplementary Materials

The following supporting information can be downloaded at: https://www.mdpi.com/article/10.3390/nu18091387/s1, Table S1: PRISMA 2020 Adherence Summary; Table S2: Metabolic and Nutritional Variables Assessed With and Without AI Support.

## Abbreviations

The following abbreviations are used in this manuscript:

AI	Artificial Intelligence
AN	Artificial Nutrition
BIVA	Body Composition Impedenzometry Assessment
IC	Indirect Calorimetry
