# Supplementary material for: Artificial Intelligence-Guided Artificial Nutrition in Critical Illness: Integrating Indirect Calorimetry and BIVA for Metabolic Precision
Source: Nutrients. 2026 Apr 28;18(9):1387. doi: 10.3390/nu18091387 (PMC13164847; doi:10.3390/nu18091387)
Supplement: Supplementary file 1 [file nutrients-18-01387-s001.zip › nutrients-4244999-supplementary.pdf]

nutrients

MDPI

Supplementary Material

The following supplementary tables have been added in response to reviewer comments.

Supplementary Table S1. PRISMA 2020 Adherence Summary

This table summarizes adherence to the PRISMA 2020 checklist for each applicable item. Items not relevant to narrative reviews are marked N/A with justification. Added in response to Reviewer 1, Comment 1.

PRISMA 2020 Item	Section	Adhered?	Notes / Justification if Not Followed
Title identifies study as a review (Item 1)	Title	Yes	Title explicitly states "narrative review"
Structured abstract with Background, Methods, Results, Conclusions (Item 2)	Abstract	Yes	All four components provided
Protocol registered (Item 24)	N/A	No	Narrative reviews are not routinely registered; design did not qualify for PROSPERO
Eligibility criteria specified (Item 5)	Methods	Yes	Inclusion/exclusion criteria defined explicitly
Information sources described (Item 6)	Methods	Yes	MEDLINE, Embase, Web of Science; 2000–2025
Search strategy reported in full (Item 7)	Methods	Yes	Full search terms listed in Search Strategy subsection
Selection process described (Item 8)	Methods	Yes	Two-reviewer screening; consensus resolution noted
Data extraction described (Item 9)	Methods	Yes	Extracted variables listed in Methods
PRISMA flow diagram (Item 17)	N/A	No	Not constructed; narrative reviews allow qualitative synthesis without PRISMA flow; selection process described textually
Risk of bias assessment (Item 12)	Methods	Partial	Formal tools not applied (not required for narrative reviews); qualitative quality evaluation performed across 4 dimensions
Effect measures and synthesis (Items 13–14)	N/A	N/A	No quantitative synthesis; qualitative narrative synthesis adopted due to study heterogeneity
Publication bias (Item 22)	N/A	N/A	Not applicable to narrative review design
Certainty of evidence (Item 15)	Discussion	Partial	Strength of evidence discussed qualitatively in Limitations section

 https://doi.org/10.3390/nu18091387

PRISMA 2020 Item	Section	Adhered?	Notes / Justification if Not Followed
Grey literature searched (Item 6)	Methods	No	Grey literature (preprints, conference proceedings, institutional reports) not systematically searched; acknowledged as limitation
Language restrictions (Item 6)	Methods	Partial	Only English-language publications retained; acknowledged as potential language bias

https://doi.org/10.3390/nu18091387

Supplementary Table S2. Metabolic and Nutritional Variables Assessed With and Without AI Support

This table was previously included in the Conclusion section (Tab 4) and has been relocated to this Supplementary section in response to Reviewer 2, Comment 2. A cross-reference has been added in the Integrated AI–IC–BIVA Framework section of the main text.

Domain	Variable	Measured Without AI	AI-Enhanced Interpretation
Energy metabolism	Resting Energy Expenditure (REE)	Indirect calorimetry (IC)	Prediction between IC measurements; trend forecasting
Substrate utilization	Respiratory Quotient (RQ)	IC-derived	Early detection of over/underfeeding patterns
Energy delivery	Q/R ratio	Manual calculation	Automated alerts and adaptive targets
Hydration status	RXc vector position	BIVA-Akern	Pattern recognition of fluid-sensitive phenotypes
Cellular health	Phase Angle (PhA)	BIVA-Akern	Prognostic stratification; trajectory analysis
Body composition	Body Cell Mass trends	Serial BIVA	Prediction of catabolic drift
Protein needs	Nitrogen balance proxies	Conventional methods	AI-driven protein titration
Metabolic phenotype	Hyper/hypometabolism	—	AI-based clustering
Nutritional risk	Under/overfeeding risk	Retrospective assessment	Real-time predictive alerts
Decision support	Nutrition prescription	—	Dynamic, patient-specific recommendations

https://doi.org/10.3390/nu18091387
